# The Cell Biology of Archaea

**DOI:** 10.1038/s41564-022-01215-8

**Published:** 2022-10-17

**Authors:** Marleen van Wolferen, Andre Pulschen, Buzz Baum, Simonetta Gribaldo, Sonja-Verena Albers

**Affiliations:** 1 Molecular Biology of Archaea, Institute of Biology II, Faculty of Biology, University of Freiburg, Freiburg, Germany; 2 Division of Cell Biology, MRC Laboratory of Molecular Biology, Cambridge, UK; 3 Evolutionary Biology of the Microbial Cell Unit, CNRS UMR2001 Department of Microbiology, Institute Pasteur, Paris, France

## Abstract

The past decade has revealed the diversity and ubiquity of archaea in nature, with a growing number of studies highlighting their importance in ecology, biotechnology, and even human health. Myriad lineages have been discovered, which expanded the phylogenetic breadth of archaea, and revealed their central role in the evolutionary origins of eukaryotes. These discoveries, coupled with advances that enable the culturing and live imaging of archaeal cells under extreme environments, have underpinned a better understanding of their biology. In this review, we focus on the shape, internal organization and surface structures that are characteristic of archaeal cells, as well as membrane remodelling, cell growth and division. We also highlight some of the technical challenges faced, and discuss how new and improved technologies will help to address many of the key unanswered questions.

## Introduction

Archaea are unicellular prokaryotic microorganisms, which at a first glance resemble bacteria in that they have no nuclear compartment or complex endomembrane systems, and have circular genomes encoding genes lacking spliceosomal introns that are often co-located in operons and co-expressed. Surprisingly however, the machineries that archaea use to replicate and express genetic information, i.e., DNA replication, transcription, and translation, are evolutionarily more closely related to those found in eukaryotes than they are to those in bacteria. At the same time, archaea also share a set of common cellular hallmarks that separate them from both eukaryotes and bacteria. These features include distinct membranes made of lipids composed of isoprenoid chains ether-linked to a glycerol-1-phosphate backbone ^1^, a unique motility apparatus called the archaellum ^2,3^, and unique types of metabolism, like methanogenesis ^4^.

**Figure 1:**
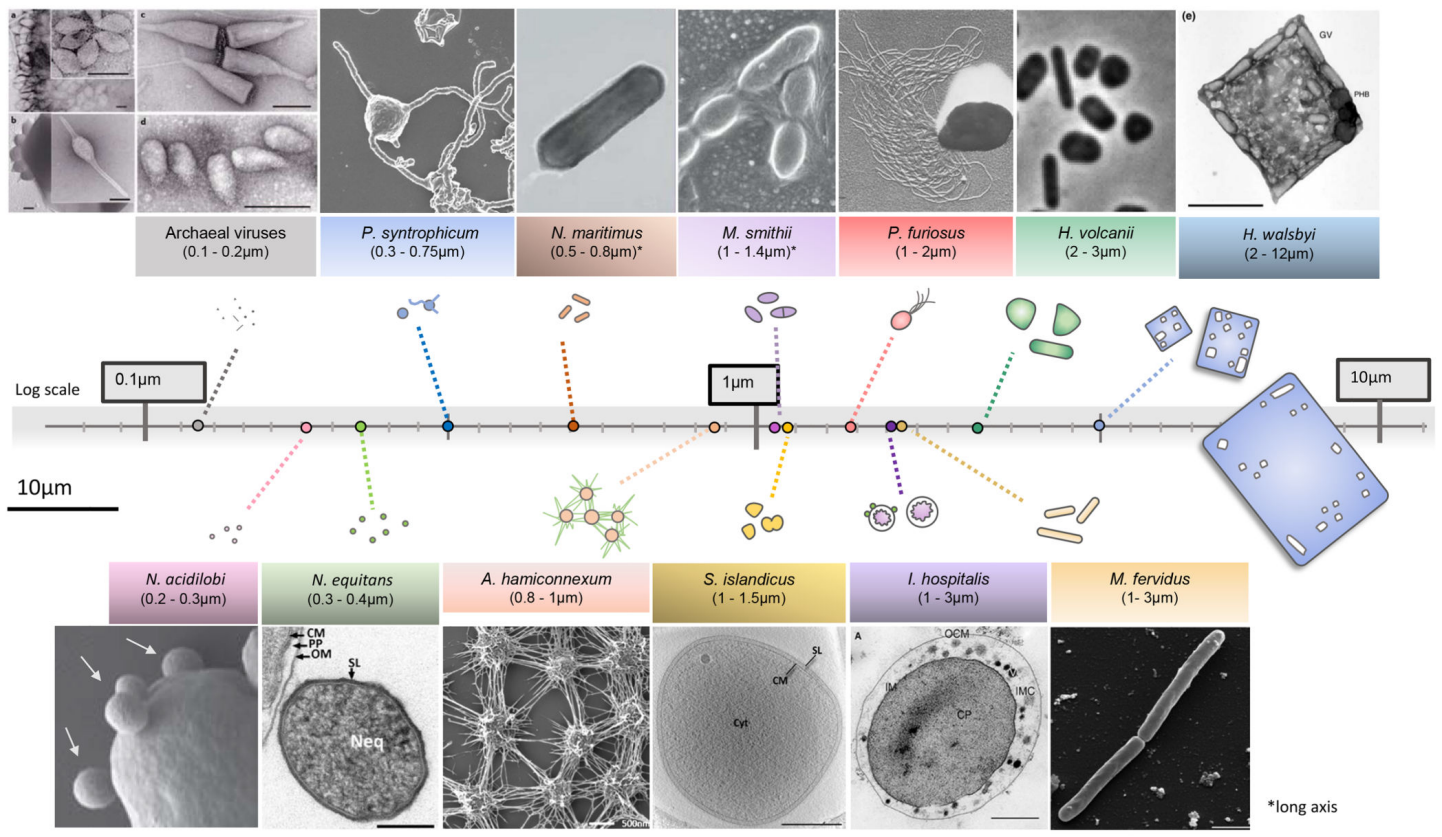
The wide spectrum of archaeal cell shapes and sizes schematically visualized in scale. Representative microscopy images are reprinted from several studies on archaea including: Archaeal viruses ^115^, *Nanopusillus acidilobi*^32^, *Nanoarchaeum equitans* ^116^, *Prometheoarchaeum syntrophicum* ^15^, *Nitrosopumilus maritimus*^117^Candidatus *Altiarchaeum hamiconnexum* ^48^, *Methanobrevibacter smithii* ^118^, *Sulfolobus islandicus* ^119^, *Pyrococcus furiosus* ^120^, *Ignicoccus hospitalis* ^19^, *Methanothermus fervidus* ^121^, *Haloferax volcanii*^23^, *Haloquadratum walsbyi* ^122^.

Archaea were initially isolated from extreme environments (e.g., high temperatures, low pH, hypersalinity), leading many to consider them as oddballs and curiosities. Over the past few years however, archaea have been found across an enormous range of environments. These environments include ocean waters and sediments, where archaea play key roles in the global nitrogen and carbon cycles ^5–7^, and in the gastro intestinal tracts of animals (including humans), where their roles and involvement in health and disease are only just beginning to be investigated ^8–10^. The use of cultivation-independent techniques to identify novel species in environmental samples has led to a rapid expansion of the phylogenetic tree of Archaea ^5–7^, emphasising their large diversity (Figure 1). For example, this enabled the identification of a diverse set of submicron-sized archaea (collectively grouped in the DPANN superphylum) from various environments, which display reduced genomes and live as obligate epibionts of different archaeal hosts ^11^. At the same time, environmental metagenomic sampling led to the discovery of a clade of uncultured archaeal lineages, the Asgard archaea, whose members are the closest prokaryotic relatives of eukaryotes ^12–14^. Strikingly, many genomes of Asgard archaea possess genes coding for homologues of eukaryotic proteins involved in activities such as cell shape control and membrane remodelling, which had never been previously found in prokaryotes. The presence of these genes, support the hypothesis that eukaryotes originated from symbiosis between a member of Asgard archaea, which gave rise to the nucleus and cell body, and the Alphaproteobacterium that eventually gave rise to mitochondria. This hypothesis was recently strengthened by the isolation of *Promethearchaeum syntrophicum*, the first member of Asgard archaea as part of a co-culture with other archaea. Many of the *P. syntrophicum* cells imaged by electron microscopy possessed long branched protrusions, possibly due to the presence of actin homologues ^15^ These appendages were seen associating with other cells in the co-culture, implying that they allow the cells to physically associate with syntrophic partners. These data make it clear that many of the molecular machines once thought to represent unique features of eukaryotic cell biology have their origins in the archaea − helping to bridge the perceived gulf that separates eukaryotes and prokaryotes.

This recent deluge of sequence data has revealed the key position that Archaea occupy in the Tree of Life. Being at the crossroad of major evolutionary events, archaea are crucial not only for understanding the origin of eukaryotes but also for our understanding of earlier events in the evolution of cellular life on Earth, notably the nature of the last universal common ancestor of all life (LUCA). As a result, archaeal cell biology is undergoing a renaissance that promises to transform these fields. In this review, we present an overview of our current knowledge of archaeal cell biology. We discuss key processes involved in cell organisation, shape, growth and division, and the archaeal cell surface. We also review the major technical challenges faced when working with archaea (Box 1, Box 2) and some open questions ahead.

### Archaeal cell organisation, shape and size

While most archaea have a simple cellular organization, with a single bounding membrane, archaeal cells come in a wide variety of shapes and sizes and can differ in their membrane architecture (Figure 1). Many archaea have a morphology like those described for common bacteria, such as cocci and rods. Others have strikingly geometric morphologies, unlike almost anything else seen in biology − forming flat squares (Figure 1) and triangles ^16^. These cell shapes arise as the result of precise cellular control of local growth and the axis of division under the guidance of cytoskeletal filaments and an overlying coat of glycosylated proteins, termed the S-layer. However, because most archaea lack peptidoglycan, these cells are also sensitive to their mechanical environment and tend to have shapes that can be easily altered by external forces.

**Figure 2:**
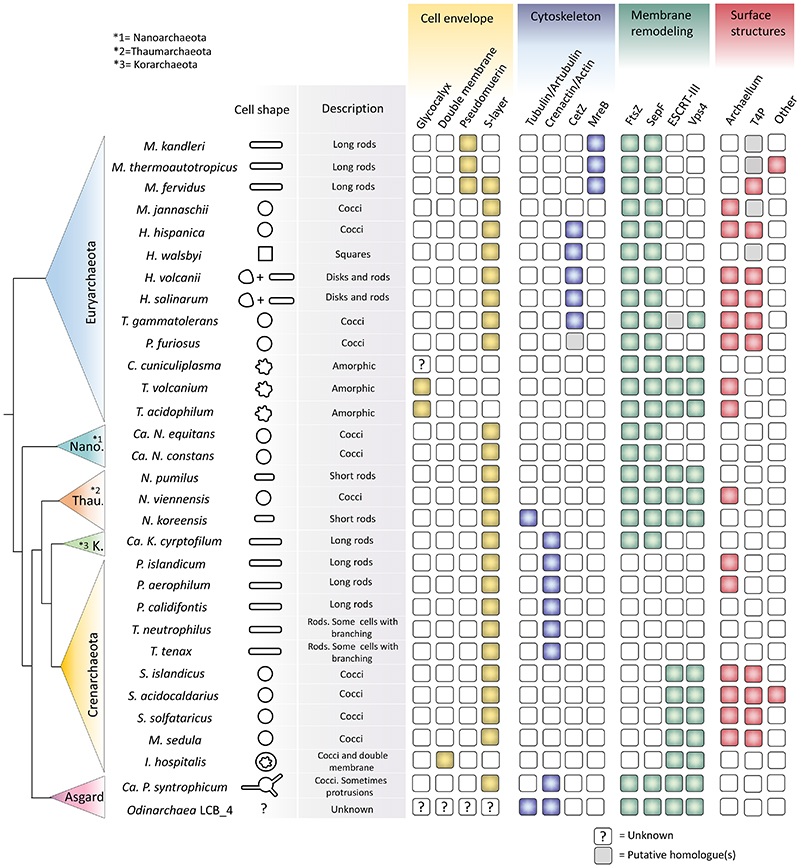
The distribution of key components of different archaeal cell biology processes. Squares filled in yellow represent the presence of different cell envelope components: glycocalyx; a double membrane; pseudopeptidoglycan; S-layer. Squares filled in blue represent the presence of cytoskeleton components: tubulin/artubulin; crenactin/actin; CetZ and MreB. Squares filled in green represent the presence of membrane remodeling components: FtsZ, SepF, ESCRT-III and Vps4. Squares filled in red represent the presence of surface structures: archaeallum, type IV pili (T4P) or other surface structures. Representative species of the following phyla have been analyzed: Euryarchaeota, Nano-archaeota (Nano), Thaumarchaeota (Thau), Korarchaeota (K), Crenarchaeota and Asgard (A).

Some archaea, like the Sulfolobales, have irregular coccoid shapes. The same is true for the vast majority of the members of the Thermoplasmatales (Euryarchaeota) and the DPANN archaea (Figure 2). Other archaea have well-defined but simple shapes, these include the majority of the Thaumarchaeaota, which tend to assume the form of short rods or cocci. In addition, members of the hyperthermophilic order Thermoproteales tend to have a thin rod shape, while others have been reported to assume branched and club-like morphologies. Among the well-studied Crenarchaeota, the members of the *Ignicoccus* genus are the structurally most striking, having no S-layer, pseudomurein or glycocalyx, but instead possessing a double membrane ^17,18^. The DNA and cytoplasm of these species are contained within an inner compartment from which membrane “tubes” emerge that connect to the overlying membrane in a manner that remains poorly defined ^19^. Remarkably, the external membrane, which is composed of a mixture of lipids and proteins, has been reported to be a site of ATP production driven via proton-motive force, implying the ability of these cells to partition different cellular activities into separate membrane compartments ^20^. Understanding how this architecture is generated and maintained promises to be a fascinating area of future research. *Ignicoccus hospitalis* also interacts with the DPANN member *Nanoarchaeum equitans*, which takes advantage of the lack of wall to form direct cell-cell connections. Moreover, it is thought that *N. equitans* cells (which lack lipid synthesis genes and are therefore obligate syntrophs) obtain their plasma membrane from the host via the exchange of lipids ^21^.

Haloarchaea are shapeshifters. For example, *Haloferax volcanii, Haloferax gibbonsii* LR2-5 and *Haloarcula japonica* are able to take on a variety of shapes under standard culture conditions, shifting from rods to flat disk-shaped cells and vice versa ^16,22^. Additionally, changes in growth conditions expands the variety of these shapes, from extremely elongated cells to very asymmetric ^22–24^. Some of these changes may be the result of specific alterations in the expression of regulators of cell shape, e.g., cytoskeletal proteins (see below). Alternatively, the extreme variability in cell shape may reflect the mechanical sensitivity of these haloarchaea since their shape changes are accentuated by slight pressure^25^.

Unfortunately, current knowledge about the morphology of the other major groups of archaea remains poor. The only representative of Korarchaeota imaged so far is the long filamentous uncultured Candidatus (Ca.) *Korarchaeum cryptofilum* ^26^. Since the *Ca. K. cryptofilum* genome, like that of members of the Thermoprotales, codes for specific archaeal actins (crenactins), actin filaments may help to generate long rod-shaped cells ^27^. Even more strikingly, the first images of *Ca. Prometheoarchaeum syntrophicum* ^15^, currently the only member of the Asgard superphylum to have been isolated in co-culture, revealed cells that are small and amorphous in shape, or can have long branched protrusions ^15^.

The sizes of archaeal cells typically vary between 0.7μm up to 4μm (the length of rods) (Figure 1), which is in the range of most bacteria. While some archaea (for example *Methanobacterium thermoautotrophicum* ^28^ and Ca. *Korarchaeum cryptofilum* ^26^) can form filaments of up to 100μm, individual cells within filaments are limited to 2-3μm in length. At the other extreme, DPANN archaea can be as small as 300nm ^29,30^. This is the case for the small spherical obligate symbiont *Nanoarchaeum equitans* ^31^ and the 100-300nm sized *Nanopusillus acidilobi*, which is found in direct contact with its host *Acidilobus* 7A ^32^. These archaea are among the smallest known living organisms ^33^, ^34^.

Despite this wide variety of cell shapes and sizes, the extent to which archaeal morphology depends on the cellular membranes, surface structure and/or cytoskeletal machinery remains to be understood, emphasizing the need for more detailed cell biological studies of a diverse set of archaea. Similarly, the impact of shape on core processes like cell growth and division remains poorly studied. However, as in bacteria, it is expected that cell shape plays a key functional role in enabling the cells to live in specific environments.

### Cell envelope

Most archaea and many bacteria have an outermost surface that is formed by a protein surface S-layer (Figure 2). Diverse surface structures can be found on this outer surface. In this section we review the S-layer and different archaeal surface structures, together with the secretion and glycosylation of surface proteins. For a more detailed review see ^35^.

#### Surface layer

**Figure 3:**
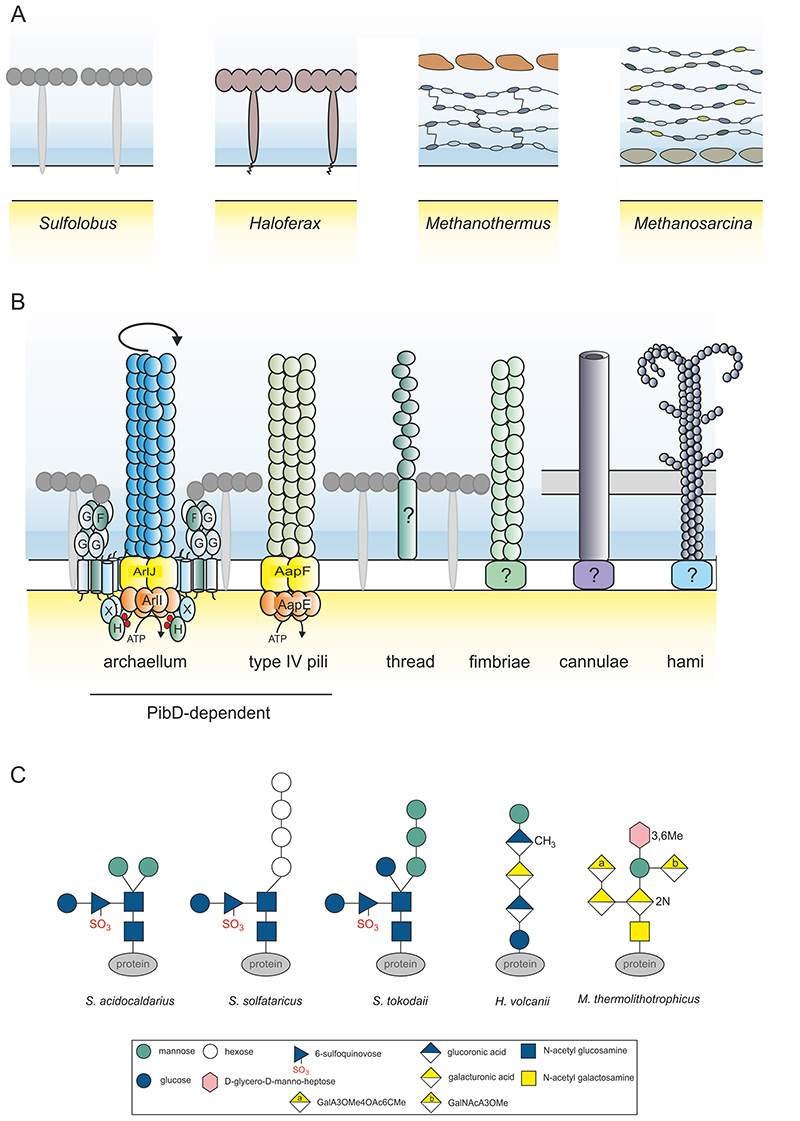
The archaeal cell envelope. (A) Examples of cell envelope structures of different archaea, including: the S-layer proteins of *Sulfolobus;* the S-layer protein of *Haloferax* containing a lipid-anchor; the cell envelope of *Methanothermus* containing both a pseudopeptidoglycan layer surmounted by an S-layer; the cell envelope of *Methanosarcina* containing an S-layer and a methanochondoitin layer on top. (B) Different archaeal surface appendages: the archaellum, type IV pili, threads, fimbriae, cannulae, and hami. (C) Different *N-*glycan structures found especially on the surface proteins of *S. acidocaldarius, S. solfataricus, S. tokodaii, H. volcanii and M. thermolithotrophicus*.

The archaeal S-layer is formed by one or two different protein subunits that self-assemble into para-crystalline lattices^36^. The subunits harbour a large lattice-forming segment and a smaller segment involved in anchoring the S-layer to the cell. These anchoring subunits are either integrated into the cytoplasmic membrane or, in certain species, into a polysaccharide layer such as pseudomurein and methanochondroitin (Figure 2, Figure 3A). The space between the membrane and the S-layer forms a quasi-periplasm that may fulfil some of the functions of the periplasm in Gram-negative bacteria ^37,38^. The archaeal S-layer subunits studied so far have been shown to be modified by *N*-linked glycosylation and, in some cases, by *O*-linked glycosylation as well ^37,39^. Although the functions of the S-layer proteins have not been studied in many archaea, S-layers are known to fulfil roles in defining the cell-shape, providing mechanical stability and functioning as molecular sieves (e.g. allowing the free passages of molecules up to a certain size depending on the pore size of the S-layer crystal) ^37,38,40,41^. It has also has been suggested that they protect cells from viruses, function in surface adhesion, and act as an anchoring scaffold for membrane proteins such as the stator complex of the archaellum (described below) ^42,43^. S-layer proteins were recently found to be dispensable for *S. islandicus* viability under laboratory conditions, but cells lacking these proteins exhibited profound defects in cell shape and size ^41,44^.

**Figure 4:**
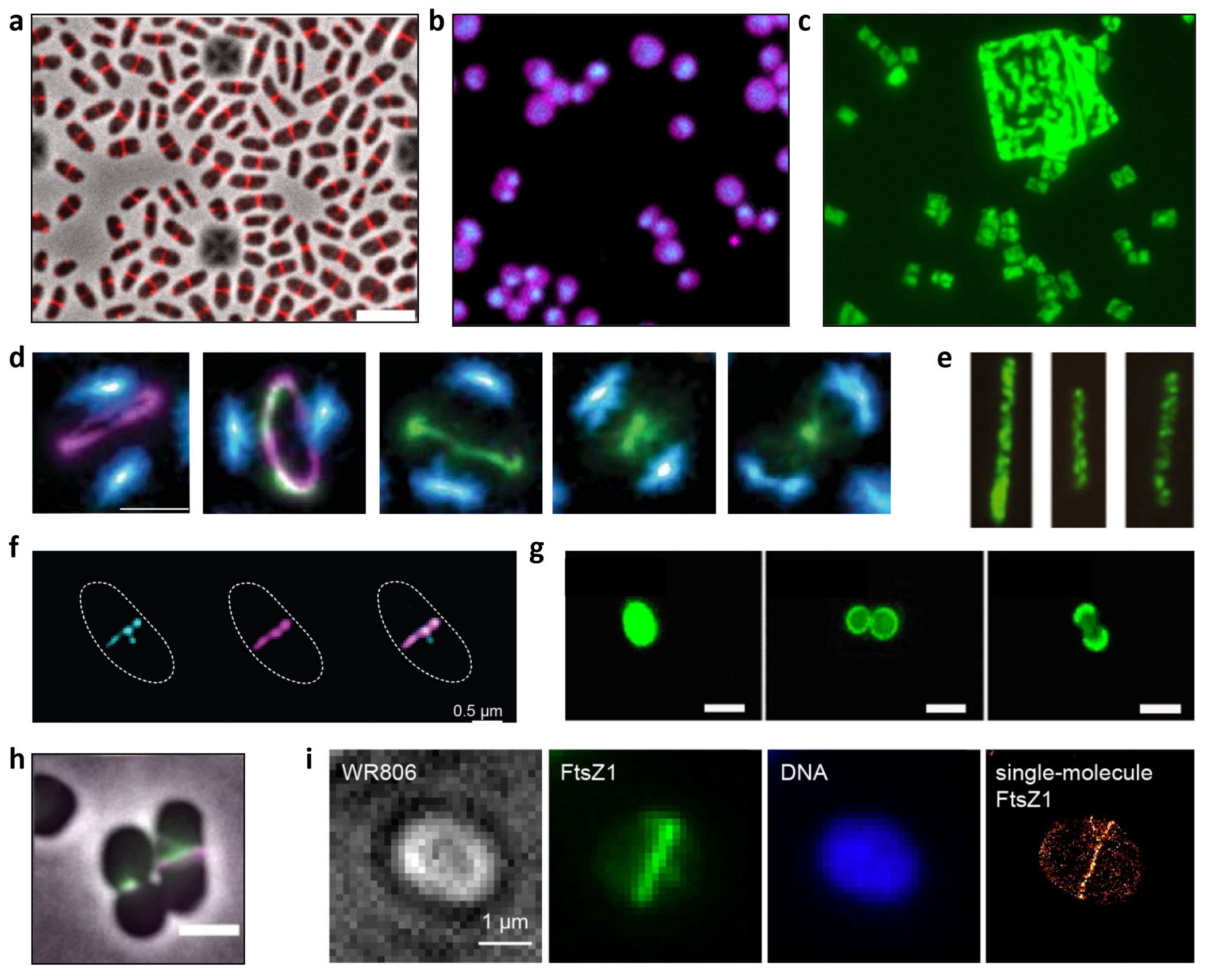
Fluorescence protein localization images from different archaeal cell biology studies. (A) Midcell localization of fluorescently tagged S-layer protein in *H.volcanii* ^23^; (B) Images of *S. acidocaldarius* with fluorescently labelled membrane (red) and DNA (blue) taken using the “Sulfoscope” at 75°C ^83^; (C) *Haloquadratum* strain Bajool9 (JCM 15065), DNA stained with acridine orange ^123^; (D) SRRF super-resolution images of immunolabeled dividing *S. acidocaldarius* cells showing ring structures of CdvB (purple), and CdvB1 (green) and DNA stained with Hoechst (blue), (scale bar, 0.5 μm) ^78^; (E) Immunolabelled *P. calidifontis* cells stained with anti-Crenactin antibodies ^27^; (F) Structured Illumination Microscopy (SIM) maximum projections of a *M. smithii* cell stained with anti-SepF (cyan) and anti-FtsZ antibodies (magenta). Scale bars 0.5 μm^102^. (G) *Pyrococcus furiosus* cell stained with Alexa Fluor 488, showing cell envelope growth (From left to right: image directly after staining; image 90 min after subculturing; and image 180 min after subculturing; scale bar, 1 μm) ^45^; (H) *H. volcanii* cells expressing fluorescently tagged FtsZ1 (purple) and FtsZ2 (green), (scale bar 2 μm) ^101^; (I) Single-molecule localization microscopy imaging of *H. volcanii* cells expressing fluorescently tagged FtsZ1 (green) ^124^.

Several questions remain regarding the assembly of S-layers in archaea, including the site of their insertion into the membrane. S-layer staining of *Pyrococcus furiosus* ^45^ (Figure 4G) and the mid-cell localization of the sole S-layer protein SLG in *H. volcanii* ^23^, suggest that they are incorporated mid cell (Figure 4A). In addition, the first atomic structure of a complete and native *H. volcanii* S-layer has recently showed how local S-layer organisation varies to accommodate changes in the curvature of the cell envelope ^36^. However, much more work is needed to determine the mechanisms coupling S-layer insertion and assembly to cell growth and division across diverse archaea.

#### Surface appendages

Archaea exhibit various surface appendages (Figure 2, Figure 3B). Species-specific appendages include cannulae, hollow tubes that connect *Pyrodictium* cells ^46^, and the hami of *Altiarchaeum hamiconexum*, which form a barbed-wire like structure that appears to anchor cells to surfaces and to one another in biofilms ^47,48^. By contrast, many archaea possess type IV pili, which are used for cell-cell and cell-surface adhesion, biofilm formation, and as anchor point for archaeal viruses ^49–51^. Their assembly pathway appears similar to that of bacterial type IV pili.

The archaellum (the archaeal flagellum) is present in all archaea capable of swimming. It is evolutionary unrelated to the bacterial flagellum, and is assembled in a manner similar to the way type IV pili are formed ^2,3^. By anchoring the archaellum motor in the S-layer, archaea are able to translate ATP-dependent motion into the torque required to propel cells forward ^43^. The proteins responsible for anchoring the archaellum in the S-layer, ArlG and ArlF ^43^, appear to have arisen as the result of duplications of the archaellum filament gene, ArlB, before being repurposed to act as stator of the archaellum motor complex ^52^.

#### Regulation of the cell surface

Similar to bacteria, most archaea are thought to use the Sec system to secrete most proteins, while a small number of cofactor-containing proteins are secreted via the TAT (twin arginine translocation) pathway, which can secrete folded proteins of sizes up to 200 kDa ^53,54^ Exceptions are the extreme halophiles, which tend to mostly use the TAT-pathway to translocate proteins in their folded state, probably to avoid protein precipitation in the presence of high salt ^55,56^. Secreted proteins are produced as pre-proteins containing signal peptides. While a Signal Peptidase I that processes the Sec-dependent and TAT secreted proteins has been described in archaea ^53^, an archaeal Signal Peptidase II, equivalent to the protein that adds lipid moieties to proteins in bacteria, has yet to be identified even though some extracellular proteins have been shown to be lipid modified at their N-termini. Similarly, while a signal recognition protein-dependent co-translational secretion system is present in archaea, it is still not clear whether archaea possess a homologue of SecA, the ATPase that provides an essential function in bacteria in driving post-translational secretion ^53^.

Only a few of the secretion systems archaea share with bacteria have been studied in depth. Most research has focused on the type IV pili assembly machinery and the homologous archaellum assembly system ^57^. Components of the machinery required to process pre-pilins and pre-archaellins are homologous to systems present in bacteria. Archaea however lack homologues of the subunits that function in bacteria to move proteins across the peptidoglycan layer and the outer membrane in Gram-negative bacteria. It has been noted, however, that the genes encoding the archaeal type IV pili assembly machinery are often found in the genomic neighbourhood of genes coding for S-layer proteins ^57^, suggesting an important interplay between S-layer and pilus assembly.

The bacterial type II secretion systems which are homologous to the type IV pili assembly machinery are used for the secretion of a range of different toxins and enzymes ^58^. In *S. solfataricus* a homologous type II secretion system, called the Bindosome, is involved in the assembly of sugar binding proteins, which play a role in sugar uptake via high affinity ABC transporters ^59^.

Type IV secretion systems are important for the transport of proteins and DNA across the membrane in bacteria ^60^. While homologous proteins have been found on archaeal conjugative plasmids, they have yet to be functionally characterized. Interestingly, a distinct DNA importer has been identified in *Sulfolobus* that has been implicated in exchanging DNA between cells to aid the repair of DNA double strand breakages^61^.

Almost all secreted archaeal proteins are modified by N- and/or O-linked glycosylation ^62^. These include S-layer proteins, substrate binding proteins, pilins and archaellins. While only a handful archaeal proteins modified through the attachment of a sugar to an asparagine residue (N-linked) have been analysed in detail, these few examples have already revealed a great variety in the size, sugar composition and degree of branching of the sugar (Figure 3C). The N-glycosylation pathways have been delineated mainly in three main model organisms: *H. volcanii, M. maripaludis* and *S. acidocaldarius*, all of which possess very different glycans and glycan assembly pathways ^62–64^. Despite these differences, all share the AglB oligosaccharyltransferase (OST). This protein is structurally and functionally homologous to SST3, the enzyme that functions at the core of the eukaryotic OST complex which transfers the sugar moiety onto a target protein^64^. Asgard archaeal genomes have also been shown to encode homologs of other components of the eukaryotic OST complex ^13^. While the functions of glycosylation are likely to be diverse in archaea, these modifications are thought to enhance the stability of extracellular proteins and to aid their translocation and folding. In addition, they have been shown to be important in haloarchaea for motility, mating and, in the context of glycosylated S-layer proteins, for species-specific cellular recognition in Sulfolobales ^37,62,64–66^.

### Internal organization

It seems likely that archaea, like bacteria, possess distinct non-membrane bound compartments where specific cellular activities are concentrated. While little is known about this type of internal organization, some of which has been attributed to the formation of molecular condensates, recent work ^67^ has pointed to asymmetries in the distribution of ribosomes, which may suggest the presence of local sites of protein synthesis or ribosomal assembly in archaea. In addition, while little is known about the location of the genome and its dynamics, the genome has been shown to be confined to only part of the cytoplasm for much of the cell cycle in *Sulfolobus* ^68^ and in *Nitrosopumulus* cells^69^ (Figure 4B).

As discussed above, the crenarchaeon *I. hospitalis* resembles Planctomycetales in possessing a complex membrane organization, with an inner membrane that is connected at points to an external membrane ^19^. The extent to which this membrane complexity is widespread in archaea has yet to be tested. Moreover, the genes responsible for this organisation have yet to be identified. It is notable, however, that many archaea possess homologues of the ESCRT-III and Vps4 machinery ^70^ which remodels membranes, for example to generate extracellular vesicles ^71^. In Asgard archaea, this machinery has been expanded through the inclusion of homologues of Ubiquitin and of ESCRT-I and ESCRT-III complex proteins, which are frequently found co-located in the genome, and which may function in targeting membrane proteins to vesicles ^72^. In addition, both members of the TACK and Asgard archaea possess homologues of additional proteins that function in membrane trafficking in eukaryotes, such as small GTPases and Sec23/24. This has led many to suggest that Asgard cells may possess sophisticated pathways of internal membrane trafficking ^13^. While this remains an exciting possibility, the rapid membrane remodelling that underpins the dynamic organisation of the eukaryotic endomembrane system also relies on other proteins, such as Dynamin, which are likely to have been inherited in eukaryotes from bacteria ^73^, together with bacterial-type lipid membranes that undergo phase separation events to assist membrane remodelling ^74^. Thus, it remains to be determined how archaea with complex membrane systems regulate their internal organization, and whether any members of the Asgard are capable of more sophisticated dynamic membrane trafficking akin to that seen in eukaryotes.

#### Genome organization

Like bacteria, archaea tend to have small circular genomes, in which genes are organized into functional groups whose transcription is often coordinated. Interestingly however, they replicate and organize their genomes in distinct and diverse ways ^75,76^. Some archaea, including euryarchaeal *Haloferax* species, have many copies of the nucleoid per cell, whereas in other organisms, such as the creanarchaeal Sulfolobales and Thaumarchaeon *Nitrosopumulus*, DNA is inherited as a single copy genome ^75,77^, making cell cycle regulation critical for survival. In case of the Sulfolobales, which possess multiple replication origins, all origins fire near synchronously, once per cell division cycle in G1, as the result of the action of a homologue the eukaryotic AAA ATPase Cdc6. Importantly, these origins do not fire again until the following cycle, in a process that appears to require proteolysis and/or cell division ^78^. Interestingly, most origins have a flanking Cdc6 homologue together with its origin recognition site − suggesting that these AAA ATPases may act locally to induce the local DNA unwinding to initiate DNA replication at the start of each cell cycle. In addition, in many of the archaea in which DNA has been imaged using specific dyes, the genome appears to be situated at a discrete site in the cytoplasm rather than having a diffused localisation − implying that it is organized. This is also suggested by HiC experiments, which show that the *Sulfolobus* genome has a higher order structure that is generated by the action of a member of the Smc condensin/conhesin family of proteins termed Coalescin. While the Coalescin loss of function phenotype has yet to be reported, it has been suggested that Coalescin contributes to global regulation of transcription as well as genome evolution ^76,79,80^. DNA binding proteins, such as Alba, Cren7 and Sul7 in *Sulfolobus* species, or histone homologues that are present in many archaea, including some Asgards ^81,82^ are also likely to influence chromosomal packing. While changes in DNA organisation can be seen in cells as they divide ^83^ little is known about the mechanisms underpinning archaeal DNA segregation. It is possible that segregation proteins such as SegA and SegB help alter DNA packing to facilitate chromosome segregation in archaea ^84,85^.

#### Archaeal Cytoskeleton

Archaea possess a variety of cytoskeletal filaments. These fall into the same classes as those found in bacteria and eukaryotes and include homologues of actin, tubulin, and ESCRT-III (Figure 2). There are reasons to think that the cytoskeletal machinery present in eukaryotes was acquired from archaea during eukaryogenesis, since the archaeal homologues are much more similar to the eukaryotic proteins than their bacterial counterparts. For example, actin is highly similar at amino acid level between Asgard archaea and eukaryotes. Archaea also possess homologues of the bacterial-type cytoskeletal families, such as: tubulin homologue FtsZ, actin homologue MreB, and PspA belonging to the ESCRT-III superfamily. For this reason, there is a pressing need to determine the dynamic behaviour of these filaments in archaea, both *in vitro* and *in vivo*, and to determine which filaments control cell shape and which regulate the changes in cell shape that accompany division and how they interact with the overlying S-layer.

The presence of actin-family cytoskeletal proteins in the genomes of archaea seems to correlate with an elongated cell shape − as it does in bacteria. Thus, as highlighted previously ^27^, rod-shaped archaeal cells (e.g., *Pyrobaculum, Methanobacter)* encode either crenactin (actin homologues that were first identified in Crenarchaeota) (Figure 4E) or MreB homologues (Figure 2) − implying a conserved function for these proteins in the regulation of cell shape across the tree of life ^86^. While immunolocalization experiments in *Pyrobaculum calidifontis* have suggested that crenactin might form a helical cytoskeletal network that spans the cell ^27^, this was inferred by analogy with helical MreB filaments in bacteria ^87^, which later proved to be an artefact of the microscopy technique ^88^. Thus, the intracellular organisation of actin filaments in archaea remains to be explored. X-ray crystallography and CryoEM studies of crenactin show that it is remarkably similar in structure to eukaryotic actin. In addition, several reports have suggested that the behaviour of these filaments may be regulated by proteins encoded by neighbouring genes in a manner similar to known regulators of eukaryotic actin filament dynamics ^89^. These observations have been used to propose that crenactin filaments may be responsible for rod cell shape and the generation of branched cell morphologies. A similar argument has been used to suggest that the actin homologues present in the genome of the Asgard *Ca. Prometheoarchaeum syntrophicum* may explain the ability of these cells to generate long protrusions, even though so far no actin filaments have been observed in these cells ^15^. Interestingly, the genomes of *Ca. P. syntrophicum* and other Asgard archaea possess genes encoding additional putative actin-regulatory proteins, including profilins and gelsolins ^90,91^, which are able to alter the dynamic behaviour of eukaryotic actin in remarkable ways *in vitro* − supporting the idea that these archaea may have a complex system of actin filament regulation.

While the correlation between the presence of MreB/actins in archaeal genomes and cell shape seems compelling, the mechanism by which these filaments dictate archaeal cell shape remains unclear. In bacteria, MreB forms curved filaments that lack a twist, which are thought to guide the path of peptidoglycan synthesis around the cell circumference to give bacteria their rod-shaped form ^92^. MreB homologues may perform a similar role in rod-shaped archaea that possess a cell wall made of pseudo-murein, an analogue of bacterial peptidoglycan ^93^. Thus, this knowledge does little to explain how actin homologues shape archaeal cells encased in a deformable S-layer lacking pseudo-murein. In a typical eukaryotic cell with a fixed elongated shape, like fission yeast, the local accumulation of actin filaments guides cell shape by helping to define regions of cell wall synthesis/removal. By contrast, in amoebae and animal cells, actin filaments work together with Myosin motors to give cells their flexible form ^94,95^, but Myosin motors have yet to be found in prokaryotes. Thus, much more research needs to be done to determine how archaeal actin and MreB homologues function to define cell shape in cells encases in an S-layer.

Many archaea also encode CetZ, a tubulin homologue ^22,24^. CetZ localizes to the periphery of *H. volcanii* cells where it appears to play an important role in the transition from a disk-shape to a rod-shape, something that profoundly changes their ability to swim ^22^. Although other archaea possess CetZ proteins, including the square archaeon *Haloquadratum walsbyi*, the spherical hyperthermophilic *Pyrococcus furiosus* and *Thermococcus gammatolerans* (Figure 2), the role of these proteins in modulating cell shape remains to be explored. In addition, the genomes of the recently isolated *Nitrosoarchaeum koreensis MY1* (Thaumarchaeota*J*) ^96^ and of the still uncultured members of *Odinarchaea* (Asgard) ^13^ encode proteins whose sequence is very similar to alpha and beta tubulin from eukaryotes ^97^. While their role has been subject to speculation, long intracellular tube-like structures have yet to be seen in such cells, and biochemical studies have yet to identify microtubules with parallel protofilaments as those found in eukaryotes and *Prosthecobacter*, a bacterium of the PVC phylum that possess tubulin homologues ^98^.

### Cell division

Cytoskeletal filaments also play critical functions in the regulation of cell division. In all cells, division requires the relatively rapid re-organisation of cellular space, mid-cell constriction and membrane scission. This is predominantly achieved using two main families of proteins implicated in archaeal cytokinesis. The first family of proteins is the tubulin homologue FtsZ which in bacteria functions to guide cell wall assembly to drive division ^99^. The second of these, the ESCRT-III family proteins perform a broadly similar function in remodelling the membrane at division to induce abscission in animal cells ^100^.

#### FtsZ based cell division

**Figure 5:**
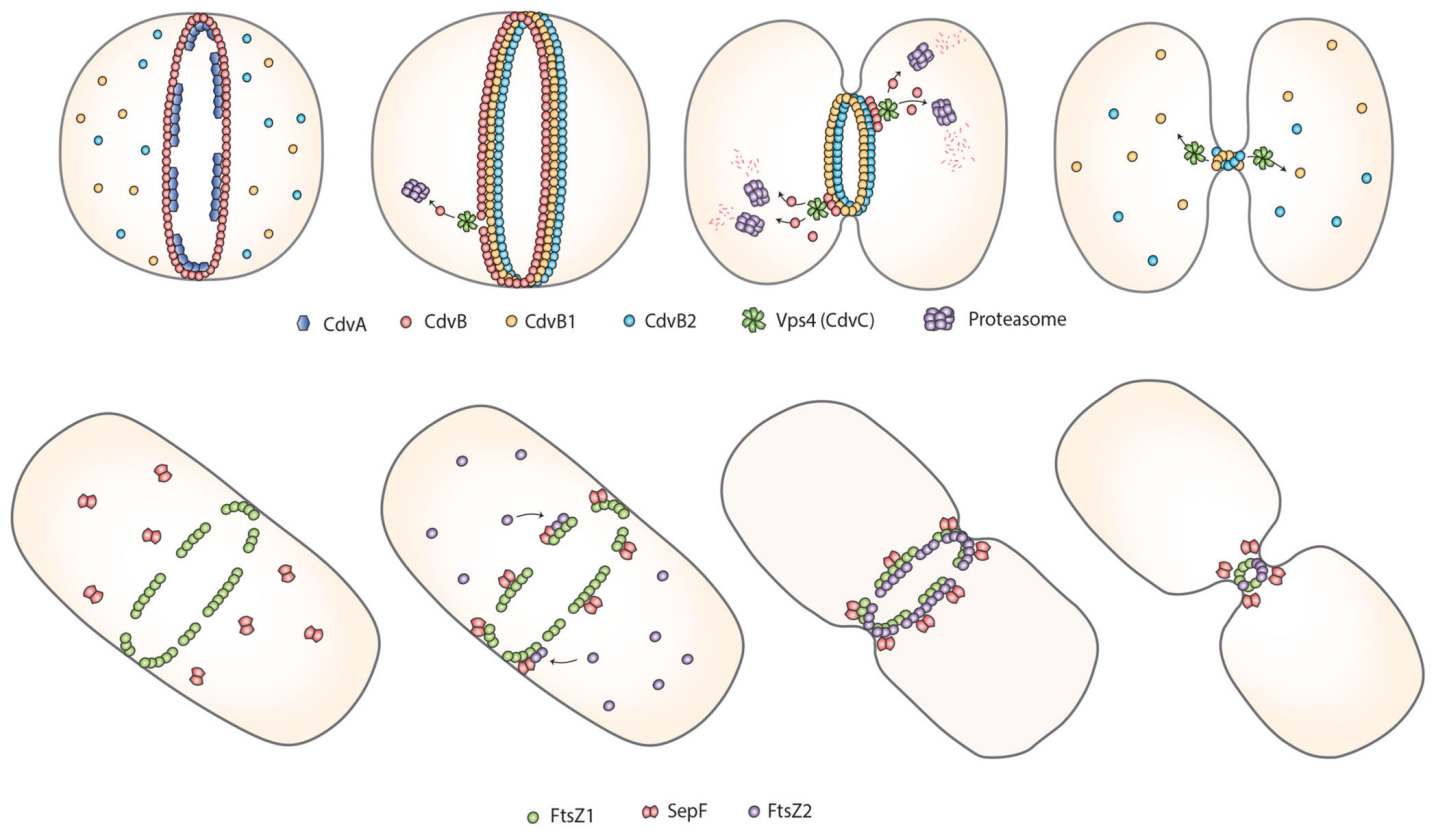
Schematic overview of the two main studied cell division mechanisms in archaea. Upper panel: the ESCRT-III mediated cell division process of *S. acidocaldarius* as displayed in four stages: (1) Localization of CdvA at mid-cell subsequently recruiting the ESCRT-III homolog CdvB, which forms a ring. (2) ESCRT-III homologues CdvB1 and CdvB2 are recruited to mid-cell by CdvB. (3) CdvB is degraded by the proteasome triggering the constriction of CdvB1 and CdvB2. (4) The Vps4 homolog CdvC most likely disassembles the CdvB1/2 ring resulting in cell membrane abscission. Lower panel: the FtsZ based cell division process of *H. volcanii* as displayed in four stages: (1) FtsZ1 forms a ring at mid-cell. (2) SepF dimers localize in an FtsZ1-dependent manner to the future cell division site, and SepF recruits FtsZ2. (3). FtsZ2 forms a ring. (4) Constriction of the divisome results in cell division.

Outside of the Crenarchaeota, most archaea possess homologues of the bacterial cell division protein FtsZ (Figure 2) ^93^. Its role in cytokinesis has been demonstrated by GFP-based localization studies in the haloarchaeon *H. volcanii*, the most well-developed euryarchaeal cell biological model. Here, FtsZ forms a ring at mid cell that contracts as the cells undergo cytokinesis ^22,101^ (Figure 5). Interestingly, most archaea possess two copies of FtsZ (named FtsZ1 and FtsZ2) ^101,102^ − which have been proposed to help the relative soft *H. volcanii* cells that lack peptidoglycan to first assemble a division ring and then divide (something resembling to the action of the archaeal ESCRT-III system mentioned below). In *H. volcanii*, both FtsZ1 and FtsZ2 form Z-rings at mid-cell (Figure 4H, I). The deletion of either protein causes severe cytokinesis defects ^101^. While the exact interplay between the two proteins remain to be elucidated, FtsZ1 appears to be involved in scaffolding the Z-ring, whereas FtsZ2 seems to be essential to initiate constriction ^101^.

More recently, the methanogen *Methanobrevibacter smithii* has been developed as a new experimental model for the study of FtsZ-based cell division ^102^. Together with its close relatives within the Methanobacteriales and Methanopyrales, *M. smithii* has two peculiar features: it possesses only one copy of FtsZ (FtsZ1), and it displays a cell wall made of pseudopeptidoglycan (pPG), making its envelope a striking evolutionary chimera of archaeal and bacterial-like features. Immunolabeled FtsZ1 showed localization to mid-cell as a discontinuous ring at the division plane in this ovococcoid archaeon. Moreover, two new Z-rings appear in the prospective daughter cells at the future division site prior to full cell constriction, similarly to the ovococcoid bacterium *Streptococcus pneumoniae*.

Recent data has added further information on the FtsZ-based cell division machinery in archaea by showing for the first time that a homologue of SepF anchors the Z-ring to the membrane, as it does in many bacteria ^102,103^ (Figure 4F). Interestingly, however, the structure of SepF in complex with FtsZ show striking differences from its bacterial counterpart; this may reflect the early evolutionary divergence of the cell envelopes in the two prokaryotic domains and/or differences in SepF/FtsZ function required to deform such very different types of cell walls ^102,103^. Curiously, and differently from bacteria, none of the MinD homologues identified in *H. volcanii* are involved in localizing FtsZ at midcell ^104^.

#### ESCRT-III based cell division

Sulfolobales are the most well-studied members of the Crenarchaeota. In *S. acidocaldarius*, homologues of ESCRT-III and the associated AAA ATPase Vps4 have been shown to drive cell division ^105,106^. First, the archaea- specific protein CdvA is thought to define the cell midzone in preparation for division. Here, CdvA initiates the formation of a non-contractile ring of the ESCRT-III homologue CdvB ^105^, which forms a template for the subsequent recruitment of two additional ESCRT-III homologues, CdvB1 and CdvB2 ^78^, which function together during division ^83^ in the form of a composite ESCRT-III ring − similar to those formed by ESCRT-III proteins in eukaryotes ^107^. CdvB is then removed from the composite ring, likely through the action of the Vps4 homologue CdvC ^105^, and is then degraded by the proteasome, allowing the composite polymeric CdvB1 and CdvB2 ring to constrict ^78^. When coupled with Vps4-dependent filament disassembly, which functions to clear the polymer from the cytokinetic bridge and which provides the energy required for membrane remodelling ^107^, this stepwise assembly and disassembly process is thought to be drive division (Figure 4D, Figure 5) ^78^. As a result, truncation mutants of the ESCRT-III proteins that cannot interact with Vps4 and cannot be disassembled have been reported to arrest division at late stages ^108^. While it is not clear why the system functions in this way, one possibility is that this multistep process enables *Sulfolobus* cells that lack a rigid wall to assemble a well-formed non-contractile ring prior to ring constriction. It should be noted that in Sulfolobales the ESCRT machinery is also involved in vesicle formation ^71,109^ and viral budding ^110,111^. Thus, as in eukaryotes, archaeal ESCRT-III proteins are able to catalyse membrane remodelling in the context of different cell biological processes.

Identifying the remaining players involved in cell division is a major goal of the next few years, as no other homologues of the bacterial divisome and elongasome are present in archaea (including those having a pPG containing cell envelope). Interestingly, *M. smithii* and its walled relatives harbour a bona fide homologue of MreB, whose role remains to be examined ^93^.

#### Other archaea

Remarkably, there are members of the Asgard and Thaumarchaeota that contain FtsZ, SepF and ESCRT-III/Vps4 homologues (Figure 2) ^69,93,102,112,113^. The presence of both division systems is intriguing, as it is not known whether they function together or perform distinct functions. Early immunolocalization experiments on the Thaumarchaeon *Nitrosopumilus maritimus*, having both ESCRTIII homologues and an FtsZ-like tubulin homologue, showed that CdvA, CdvB and CdvC localize to mid-cell between segregated nucleoids as they do in *Sulfolobus* whereas the FtsZ-like tubulin homologue appeared to have a diffuse localisation making its role in cytokinesis unclear ^69^. Finally, members of the *Thermoprotales* do not seem to encode any FtsZ or ESCRT-III proteins (Figure 2). It has been proposed that they divide through the action of the crenactin cytokskeleton and of the actin-related arcadins ^27^ − perhaps in a similar manner as MreB functions in division in some bacteria that lack FtsZ ^114^.

### Outlook

In this review we have described the state of the art of archaeal cell biology and discussed their morphologies, cellular organization, cell envelope and internal organization. This already makes clear the great diversity of fundamental cellular processes present in archaea and hints at the biological novelty that remains to be unearthed.

As is seen for bacteria, archaea have a wide variety of cell-shapes, yet it remains unclear to what extent archaeal cell morphology depends on the cellular membranes, surface structure and/or cytoskeletal machinery. Similarly, the impact of shape on cell growth and division is poorly understood. Future and more detailed cell biological studies on diverse and newly discovered archaea will help answer these questions. Another major goal will be to characterize the mechanisms underlying the highly diverse archaeal cell division machineries. These studies will not only illuminate fundamental aspects of archaeal cell biology, but also provide key data to understand the links between cellular elements (growth, division, cell surface, motilityand surface structures) and the functioning of archaea in their diverse natural environments, including the human microbiome ^9^.

Achieving these research goals will require overcoming the technical challenges faced when studying archaea. Ongoing advances in high-resolution fluorescent thermo-microscopy, and the development of thermostable fluorescent proteins, will help investigating outstanding questions on the dynamic cellular processes of thermophilic archaea (Box 1). Moreover, the development of culturing conditions and genetic systems (Box 2) for additional archaeal representatives, especially members of the Asgard archaea, will contribute greatly to our understanding of the diversity of fundamental cellular processes across all archaea. This will in turn provide crucial insights into the characteristics of the LUCA and processes that led to eukaryogenesis.

## Box 1 Microscopy techniques

Microscopy has contributed greatly to our understanding of archaeal cell shape, cell division, motility, virus infection and vesicle formation. Immunostaining of fixed cells for instance has allowed the visualization of: FtsZ in *Haloferax mediterranei* ^125^; Cdv-proteins in *Sulfolbus acidocaldarius* ^105,106^*;* SepF in *Methanobrevibacter smithii* ^102^, crenactin in *Pyrobaculum calidifontis* ^27^ and archaeal exosomes in *Sulfolobus solfataricus* ^126^. However, to give more insights into the dynamics of cellular processes, live-imaging is required. In extremophilic archaea this has been challenging because of the technical limitations at their living conditions, especially high temperatures. Currently there are a few thermo-microscopy setups which have enabled the visualization and analysis of swimming *S. acidocaldarius* cells ^127–131^ and cell division of *Pyrodictium abyssii* ^132^ and *S. acidocaldarius* ^83,133^ at their natural growth temperatures. Among these imaging setups, the *“Sulfoscope”* combines thermo- with fluorescence microscopy ^83^. It thereby allows for instance the visualization of fluorescently labelled DNA, giving new insights into the coordination and organization of DNA condensation and segregation. In addition, it may in the future be possible to grow cells in microfluidic devices, which prevent evaporation. In addition to high resolution thermo-microscopy, there is a need for diverse thermostable fluorescent probes allowing the localization of proteins in living cells, as only a few are currently available ^134,135^. Optimizing and expanding the range of these proteins will enable the localization of dynamic proteins in thermophilic archaea. In contrast, live cell imaging is far more advanced for mesophilic haloarchaea ^25^. Different functional fluorescent proteins adapted to high salt concentrations have enabled the localization and study of several dynamic cellular processes in *H. volcanii* ^93^. Recently, the first single-molecule localization microscopy experiments were performed in archaea. For that, an autofluorescence-free *H. volcanii* strain was constructed and new fluorescent proteins were developed ^124^. Together, ongoing developments in the field of archaeal microscopy promise the generation of several new high-resolution images of archaea and their sub cellular structures at their natural growth conditions.

## Box 2 Genetic tools

Applications of genetic systems in cell biology include the phenotypic analysis of gene deletion and expression strains, and the fluorescent labelling and localization of proteins of interest. Despite the availability of several culturable archaeal strains, the lack of selection markers has long hampered the development of genetic tools. In fact, many of the commonly used antibiotics in bacteria either do not have targets in archaea or are degraded at the extreme conditions in which many archaeal model organisms live ^136,137^. The first archaeal genetic tools were established in the 1990s for the methanogenic *Methanoccocus maripaludis* and *Methanosarcina barkeri* ^138,139^ and for the halophilic *Halobacterium salinarum*, *H. volcanii* and *H. mediteranii* ^140–143^. Soon after that, genetic systems were developed for the (hyper)thermophilic: *Thermococcus kodakarensis*, *Pyrococcus furiosus* ^144,145^ and Sulfolobales ^146–148^. Over the last decades several other archaeal genetic systems based on auxotrophic markers as well as (archaea-specific) antibiotics have been established ^149^. With the growing interest in archaea, the number of model organisms and successfully established genetic systems are continuously expanding. Several methods using selection and counter selection marker genes that allow the deletion, exchange or insertion of DNA have now been used. Auxotrophic markers used in these methods include genes that play a role in metabolic pathways, for instance those for pyrimidine, histidine, and tryptophan. Functional antibiotics include: novobiocin (inhibiting DNA gyrase), simvastatin (inhibiting 3-methylglutaryl coenzyme A), puromycin and hygromicin (inhibiting translation). Next to the classical gene deletion and expression approaches, novel methods include the application of CRISPR-Cas for gene silencing (CRISPRi) and genome editing ^150^. These available genetic tools have provided many insights into different topics in the field of archaeal cell biology including cell shape, cell division, chromosome organization, surface structures, S-layer function. With these ongoing developments many more questions regarding the cell biology of the archaeal cell will be tackled.
